# A rapid, non-invasive tool for periodontitis screening in a medical care setting

**DOI:** 10.1186/s12903-019-0784-7

**Published:** 2019-05-23

**Authors:** Martijn J. L. Verhulst, Wijnand J. Teeuw, Sergio Bizzarro, Joris Muris, Naichuan Su, Elena A. Nicu, Kamran Nazmi, Floris J. Bikker, Bruno G. Loos

**Affiliations:** 1 Department of Periodontology, Academic Centre for Dentistry Amsterdam (ACTA), University of Amsterdam and VU University, Gustav Mahlerlaan 3004, 1081 LA Amsterdam, the Netherlands; 2 Department of Comprehensive Dentistry, Academic Centre for Dentistry Amsterdam (ACTA), University of Amsterdam and VU University, Gustav, Mahlerlaan 3004, 1081 LA Amsterdam, the Netherlands; 3 Department of Social Dentistry, Academic Centre for Dentistry Amsterdam (ACTA), University of Amsterdam and VU University, Gustav Mahlerlaan 3004, 1081 LA Amsterdam, the Netherlands; 4 Dental Clinic CMI Dr. Opris M.I, Str. Nicolae Iorga, 40 Sibiu, Romania; 5 Department of Oral Biochemistry, Academic Centre for Dentistry Amsterdam (ACTA), University of Amsterdam and VU University, Gustav Mahlerlaan 3004, 1081 LA Amsterdam, the Netherlands

**Keywords:** Periodontitis, Screening, Prediction model, Self-reported oral health, Questionnaire, Salivary biomarkers

## Abstract

**Background:**

Since periodontitis is bi-directionally associated with several systemic diseases, such as diabetes mellitus and cardiovascular diseases, it is important for medical professionals in a non-dental setting to be able examine their patients for symptoms of periodontitis, and urge them to visit a dentist if necessary. However, they often lack the time, knowledge and resources to do so. We aim to develop and assess “quick and easy” screening tools for periodontitis, based on self-reported oral health (SROH), demographics and/or salivary biomarkers, intended for use by medical professionals in a non-dental setting.

**Methods:**

Consecutive, new patients from our outpatient clinic were recruited. A SROH questionnaire (8 questions) was conducted, followed by a 30 s oral rinse sampling protocol. A complete clinical periodontal examination provided the golden standard periodontitis classification: no/mild, moderate or severe periodontitis. Total periodontitis was defined as having either moderate or severe. Albumin and matrix metalloproteinase-8 concentrations, and chitinase and protease activities were measured in the oral rinses. Binary logistic regression analyses with backward elimination were used to create prediction models for both total and severe periodontitis. Model 1 included SROH, demographics and biomarkers. The biomarkers were omitted in the analysis for model 2, while model 3 only included the SROH questionnaire. The area under the receiver operating characteristic curves (AUROCC) provided the accuracy of each model. The regression equations were used to create scoring algorithms, composed of the remaining predictors, each with its own weight.

**Results:**

Of the 156 patients participating in this study, 67% were classified with total periodontitis and 33% had severe periodontitis. The models for total periodontitis achieved an AUROCC of 0.91 for model 1, 0.88 for model 2 and 0.81 for model 3. For severe periodontitis, this was 0.89 for model 1, 0.82 for model 2 and 0.78 for model 3. The algorithm for total periodontitis (model 2), which we consider valid for the Dutch population, was applied to create a freely accessible, web-based screening tool.

**Conclusions:**

The prediction models for total and severe periodontitis proved to be feasible and accurate, resulting in easily applicable screening tools, intended for a non-dental setting.

**Electronic supplementary material:**

The online version of this article (10.1186/s12903-019-0784-7) contains supplementary material, which is available to authorized users.

## Background

Periodontitis is a complex, chronic inflammatory disease due to an aberrant host response to bacteria in the dental biofilm. In susceptible individuals, this destructive disease affects the supporting structures of the teeth (root cementum, periodontal ligament and alveolar bone), resulting in loosening of teeth and eventually tooth loss [1]. Periodontitis is estimated to affect approximately 40% of the adult population, with 8–11% suffering from the severe form [2–4].

For the past several decades, the relationship between periodontitis and general health has received considerable attention. Not only is periodontitis more prevalent in patients with other chronic diseases, such as rheumatoid arthritis [5] and diabetes mellitus [6], evidence has accumulated that periodontitis may be another and additional risk factor for atherosclerotic cardiovascular diseases [7, 8], adverse pregnancy outcomes [8] and diabetes mellitus [9]. In the case of diabetes mellitus, patients do not only have an increased risk for the onset and/or progression of periodontal disease, established periodontitis can also negatively influence metabolic control [9]. This urged the International Diabetes Federation (IDF) in 2009 to publish oral health guidelines for diabetes care professionals [10]. In 2013, the Dutch college of general practitioners (NHG) followed these recommendations by including an advice on oral health in the national diabetes care guidelines, with special focus on periodontitis during the annual diabetes check-up: *“The family physician inspects the mouth, and pays attention to signs of periodontitis. He advises the patient to visit the dentist/oral hygienist twice a year”* [11]. Although the publication of the advice in the guideline is justified, it is not followed-up in daily practice. Limited consultation time and a lack in resources and specific knowledge do not allow thorough inspection of the oral cavity. Consequently, family physicians and nurse practitioners are often not able to recognize the signs for periodontitis as recommended in the guidelines. It is highly conceivable that this will also be the case for other medical specialists, such as cardiologists and internists. To facilitate the medical community aims to pay attention to periodontitis in a primary care and/or hospital setting, a ‘quick and easy’, non-invasive screening method without dental inspection is preferred.

In the United States, a self-reported oral health (SROH) questionnaire was developed and validated to screen for periodontitis [12–14]. The questionnaire consisted of eight items, and was recommended in the IDF guidelines on oral health [10]. However, this questionnaire did not yet provide care professionals with a clinical tool for easy implementation in their daily practice. The next step in facilitating implementation in daily care practices is the development of a screening tool which converts these individual questions into one single score that predicts whether the patient is likely to suffer from periodontitis or not. A recent study from France developed such a self-reported screening score for severe periodontitis, partly based on the same questions used in the study from the United States [15]. Both studies emphasize the importance of national validation of a screening tool based on SROH, since it could be region and culture dependent.

In addition to SROH, it is envisaged that biomarkers in oral fluids – from this point on referred to as salivary biomarkers – could also be used to screen for periodontitis. Diabetes care professionals are already familiar with point-of-care sampling and analysis of plasma biomarkers. Salivary biomarkers could therefore be a feasible addition to their daily practice, provided that a quick and easy, non-invasive sampling method is used to minimize the burden. However, despite substantial and promising research in this field, widespread clinical implementation of salivary biomarkers as a screening tool has not been achieved so far. Indeed, several biomarkers related to periodontitis have been identified, but it is unlikely that a single biomarker will reach the required sensitivity and specificity to serve as a reliable screening tool [16, 17]. However, in combination with SROH and demographic data, certain salivary biomarkers could have a valuable contribution to predicting periodontitis.

Therefore, this study’s primary objective is to develop and validate a clinical tool, based on a combination of SROH, demographics and salivary biomarkers, which can be used to screen for periodontitis in a medical care setting. Given that in some cases, there might be practical reasons to omit collection of oral rinse samples, we will also assess the predictive performance of the clinical screening tool without salivary biomarkers.

## Methods

For this study, newly admitted consecutive patients (≥18 years of age) at the Academic Centre for Dentistry Amsterdam (ACTA) were recruited while attending for the first time the outpatient clinic. There was no (recent) dental or medical information available about these patients beforehand. The study was approved by the medical ethical committee of the Vrije Universiteit Medical Centre (2014.585 [A2016.155]). All patients with at least one natural tooth were eligible; edentulous patients, with or without full dentures, were not considered (regardless of dental implant support). First, the SROH questionnaire was conducted, followed by the oral rinse sampling. Next, a complete clinical periodontal examination was performed by one of two calibrated periodontists (SB and WJT). Finally, demographic data, such as age, sex and smoking status were derived from the electronic health records.

### Self-reported oral health questionnaire (SROH)

The original SROH questionnaire consisted of eight items [14]. The questions, their Dutch translations and the used abbreviations can be found in Additional file 1. The questionnaire was conducted by a non-dentist (author MJLV), mimicking the setting where non-dental medical professionals conduct the questionnaire. All questions were closed-ended; five items had ‘yes’ or ‘no’ as answer possibilities (Q1 and Q3 – Q6). In one item (Q2), the patient was asked to rate the health of his/her teeth and gums on a five-point scale. Question 7 and 8 inquired how often the patient used interdental hygiene products (Q7) or mouthwash/oral rinse products (Q8), expressed as days per week. Finally, each item had an additional answer possibility of ‘don’t know’, and the patient was also allowed to refuse to answer if desired.

### Oral rinse sampling

To collect the oral rinses, a sampling and processing method was used as described previously [18]. The following minor adjustments were made to make the method more suitable for the primary care setting. Instead of the rather time-consuming protocol of three times 30 s rinsing with 20 ml phosphate buffered saline (PBS) – which also tastes rather bitter – the patient was instructed to thoroughly rinse once with a more user-friendly 10 ml saline solution (0.9% sodium chloride) for 30 s, as used before for the characterization of oral neutrophils [19]. In order to create realistic circumstances comparable to daily practice in a non-dental setting, there were no pre-defined requirements for the participants regarding the condition of the oral cavity (e.g. tooth brushing or time after having a meal). The patients were instructed to swallow once before starting the 30 s oral rinse protocol. After expectorating in a medicine cup, the sample was transferred back into a coded 50 ml Falcon tube and stored on ice for a maximum of three hours. Later, the samples were resuspended by vortexing for several seconds, and filtered, to remove any food residue and epithelial cells. Filters with a pore size of 70 μm (EASY Strainer™, Greiner Bio-One GmbH, Frickenhausen, Germany), 30 μm and 10 μm (both Merck Millipore™ Ltd., Cork, Ireland) were used in that specific order. Next, the filtered samples were centrifuged at 500 *g* at 4 °C for 10 min. The supernatant was aliquoted and stored at − 80 °C until further use.

### Periodontal examination

Clinical periodontal examination was performed by one of two calibrated periodontist (SB or WJT). The examination consisted of measurements of both positive and negative gingival recessions, probing pocket depth (measured from the margin of the gingiva to the depth of the pocket) and bleeding on probing (presence/absence) at six sites per tooth. The Centers for Disease Control and Prevention-American Academy of Periodontology (CDC-AAP) case definition for periodontitis was used: *Severe periodontitis*: the presence of 2 or more interproximal sites with ≥6 mm attachment loss (not on the same tooth) and 1 or more interproximal site(s) with ≥5 mm probing pocket depth; *Moderate periodontitis*: 2 or more interproximal sites with ≥4 mm clinical attachment loss (not on the same tooth) or 2 or more interproximal sites with probing pocket depth ≥ 5 mm, also not on the same tooth); *Mild* or *no periodontitis*: neither “severe” nor “moderate” periodontitis. *Total periodontitis* represents all patients with moderate or severe periodontitis [20].

### Biomarker analysis

#### Albumin

Albumin levels were analyzed using Enzyme-Linked Immuno Sorbent Assay (ELISA). To do so, 96-wells microplates were coated overnight at 4 °C with 100 μl polyclonal rabbit anti-(human albumin) antibody (DAKO, Denmark, Glostrup) in 100 μl coating buffer (Na_2_CO_3_; pH 9.6). Next, the oral rinse samples were diluted 1:100 in PBS supplemented with 0.1% Tween-20 (PBS-T), and added to the microplates in duplicate. As standard, human serum albumin solution was used. Both the standard and the samples were serially diluted twofold in PBS-T and incubated for 1 h at 37 °C. Next, horseradish peroxidase-conjugated rabbit anti-(human albumin) (GeneTex, Irvine, CA, USA) was added, followed by one hour of incubation at 37 °C. Finally, O-Phenylenediamine dihydrochloride solution was used as substrate, and the reaction was stopped by adding 50 μl of 4 N H_2_SO_4_ solution. The final albumin concentration (μg/ml) was measured using a Multiskan™ FC Microplate Photometer (Thermo Scientific Multiskan FC, Rockford, IL, USA).

#### Chitinase activity

Chitinase activity was measured by adding 90 μl oral rinse sample to 10 μl substrate solution of 4-Methylumbelliferyl β-D-N,N′,N′′-triacetylchitotrioside (Sigma-Aldrich Chemie B.V., Zwijndrecht, Netherlands) in black 96-wells microplates (Greiner Bio-One GmbH, Frickenhausen, Germany) [21]. All microplates contained a reference sample that consisted of pooled oral rinses, collected from 10 healthy dental students and staff members. All samples were analyzed in duplicate. Chitinase activity, expressed as increase in fluorescence, was measured for 1 h at 37 °C using a microplate reader (BMG FLUOstar Galaxy, MTX Lab Systems, Vienna, VA, USA). The chitinase activity for the reference sample was set as 1 arbitrary unit (AU)/ml; all oral rinse samples were quantified relative to the reference sample.

#### Protease activity

Protease activity in the oral rinses was determined using black 96-wells microplates (Greiner Bio-One GmbH, Frickenhausen, Germany). In each well, 80 μl oral rinse sample was added, together with 20 μl of PEK-054 ([FITC]-NleKKKKVLPIQLNAATDK-[KDbc]), a substrate previously used to assess total protease activity [22]. A trypsin solution from bovine pancreas (500 U start concentration) was used as standard, which was serially diluted in two-fold steps. The samples and standards were analyzed in duplicate. Protease activity, expressed as increase in fluorescence, was measured continuously at 37 °C using the BMG FLUOstar Galaxy microplate reader (MTX Lab Systems), and terminated when no further increase was observed (after approximately 1 h).

#### MMP-8

The MMP-8 concentrations in the oral rinse samples were analyzed using a commercially available pre-coated human MMP-8 ELISA kit (Boster Biological Technology, Pleasanton, CA, USA) according to the instruction manual provided by the manufacturer.

### Statistical analysis

IBM SPSS statistics version 25 was used for statistical analysis. Similar to the original study [14], items from the SROH questionnaire with more than 2 outcome possibilities were dichotomized, and all responses where coded with either 0 or 1. Further, as described previously, missing and refused items, as well as the response ‘don’t know’, were excluded from analysis [14]. Age was also recoded into two groups: younger than 40 years old vs. 40 years or older.

Next, Shapiro-Wilk tests were used to assess whether the continuous variables (clinical measurements and biomarkers) followed a normal distribution. Descriptive statistics were applied to provide insight in patient characteristics. Next, univariate associations between the potential predictors and periodontitis were assessed. One-way ANOVAs or Kruskal-Wallis tests were used to analyze continuous data (clinical measurements and biomarkers) across all periodontitis groups (no/mild, moderate and severe periodontitis). Student’s t-test or Mann-Whitney U tests were used to test for differences between *total periodontitis* and *no/mild periodontitis*, and between *severe periodontitis* and *no severe periodontitis.* Chi-square tests were used to analyze categorical data (SROH and demographics).

To prevent overfitting of the prediction models, only candidate predictors that showed a univariate association with total and/or severe periodontitis were selected for the prediction modelling, with a cut-off *p*-value of 0.20 [23]. Each of the predictors selected for modelling was tested for multi-collinearity through assessment of the variance inflation factor (VIF) and tolerance, by running linear regression analysis. VIF values higher than 5 and/or tolerance values lower than 0.1 indicated serious suspicion of multicollinearity, as suggested in literature [24, 25].

Clinical screening tools were developed for two specific disease outcomes: total periodontitis (moderate and severe combined) and severe periodontitis. This consisted of five steps:

#### Step 1: modeling

Prediction models were developed by performing binary logistic regression analysis. For both disease outcomes (total periodontitis or severe periodontitis), three models were created and assessed. Model 1 included all candidate predictors in the analysis. The biomarkers were omitted in the analysis for model 2, while model 3 only included the SROH questionnaire. Total and severe periodontitis were entered as dependent variables, while candidate predictors – determined with the univariate analysis – were entered as covariates. Stepwise backward elimination by likelihood ratio removed the predicting items with the highest *p*-value from the models step by step, until all of the remaining items had a statistically significant contribution. The predicted probability was saved as a new variable.

#### Step 2: discrimination

The discriminative performance – or accuracy – of the created models could be described as the ability to distinguish between subjects with and without the disease outcome, in this case (severe) periodontitis. The accuracy was expressed as the area under the receiver operating characteristic (ROC) curve (AUROCC), also known as c-statistic [26]. This was derived by plotting the predicted probability (derived during step 1) against the actual disease state in an ROC curve. An AUROCC of 1.0 indicates that the model perfectly discriminates between diseased and non-diseased subjects; an AUROCC of 0.5 means the model doesn’t discriminate better than ‘random’ (i.e. flipping a coin) [26]. For further analysis, the optimal predicted probability cut-off value had to be identified. This predicted probability was defined as the value with the highest sum of sensitivity and specificity across the ROC curve.

#### Step 3: calibration

Agreement between the predicted probability and observed probability (the prevalence of total or severe periodontitis) was assessed by calculating the Hosmer-Lemeshow goodness of fit statistic. A *p* > 0.10 indicated that the model fitted the data [27].

#### Step 4: clinical values

A new binary variable was computed, which represented the predicted disease outcome by classifying each individual as either diseased or non-diseased, based on the cut-off score derived in step 2. By creating a two-by-two contingency table with the predicted disease outcome versus the actual disease state, several clinical values were calculated (sensitivity, specificity, positive and negative predictive value).

#### Step 5: scoring method

An individual *sumscore* was composed by expressing the prediction model with the following formula:

$$ Y={B}_1\ast {X}_1+{B}_2\ast {X}_2\dots {B}_n\ast {X}_n $$

In this formula, the sumscore (Y) was composed by the predictors (X) – that remained after the binary logistic regression analysis – which all had their own weight, expressed as a constant value (B [regression coefficient]). For the binary predictors, a reference outcome was set a priori by coding a negative outcome as 1, and a positive outcome as 0. For the biomarkers, no reference outcome was required. The sum of responses to all predictors – multiplied with their individual weight (B) – resulted in an individual sumscore. The sumscore that corresponded with the predicted probability cut-off value determined in step 2 was identified. This score represented the threshold value from which the model classified an individual as (severe) periodontitis patient.

The algorithm for total periodontitis using SROH and demographics (model 2) will be applied to develop a freely accessible, web-based screening tool.

## Results

### Patient characteristics

A total of 156 patients were included in this study; 51 (32.7%) were identified with severe periodontitis, 54 (34.6%) with moderate periodontitis and 51 (32.7%) patients had mild or no periodontitis. Five patients had one or more dental implants in between natural teeth. Demographic and other patient characteristics are presented in Table 1. The mean age of the total population was 45.2 ± 16.4 years, with 55.1% being male and 23.7% being smoker. Patients with periodontitis – both total and severe – were significantly older, had fewer teeth and were more likely to be male and smoker, compared to patients without (severe) periodontitis. The values for the clinical parameters, bleeding on probing, probing pocket depth and clinical attachment loss, were significantly higher with increasing severity of periodontitis.

**Table 1:** Demographic, dental and periodontal characteristics for the study population (*n* = 156)

	No or mild periodontitis^d^	Moderate periodontitis^d^	Severe periodontitis^d^	Total periodontitis (moderate + severe)^d^
Demographics				
* N* (%)	51 (32.7)	54 (34.6)	51 (32.7)	105 (67.3)
Age (years)	33.2 ± 13.9	48.1 ± 15.7	54.1 ± 11.8^b^***	51.0 ± 14.2^a^***
Sex				
Male	22 (43.1)	27 (50.0)	37 (72.5)^b^**	64 (61.0)^a^*
Female	29 (56.9)	27 (50.0)	14 (27.5)	41 (39.0)
Smoking				
Yes	8 (15.7)	9 (16.7)	20 (39.2)^b^**	29 (27.6)^c^
No	42 (82.4)	41 (75.9)	30 (58.8)	71 (67.6)
Missing	1 (2.0)	4 (7.4)	1 (2.0)	5 (4.8)
Clinical measurements				
Number teeth	28.1 ± 3.81	24.3 ± 4.46	24.1 ± 4.64^b^**	24.2 ± 4.53^a^***
Bleeding on probing (%)	24.5 ± 15.7	29.2 ± 17.9	46.6 ± 26.2^b^***	37.6 ± 23.9^a^***
Probing pocket depth (mm)	2.12 ± 0.26	2.31 ± 0.34	3.18 ± 0.88^b^***	2.73 ± 0.79^a^***
Clinical attachment loss (mm)	1.36 ± 0.45	2.08 ± 0.63	3.90 ± 1.37^b^***	2.96 ± 1.39^a^***

Data are presented as either mean ± SD or n (%)

Mann-Whitney U tests (for continuous data) and chi-square tests (for categorical data) were used to assess differences between groups:

^a^significantly different from no/mild periodontitis

^b^significantly different from patients without severe periodontitis

^c^Although not reaching statistical significance, the variable did suffice the p < 0.20 cut-off level to be included in the regression analysis

^d^definitions for periodontitis according to Page and Eke (2007) [20]

*p < 0.05, **p < 0.01, ***p < 0.001

### Self-reported oral health

The answers to the SROH questionnaire can be found in Table 2. The response rate was well above 95%, except for ‘gum disease’ (Q1), which had 16 missing responses (10.3%). With the exception of question on ‘floss use’ (Q7) and ‘mouthwash use’ (Q8), the other six questions were significantly associated with both total and severe periodontitis. In the case of severe periodontitis, all questions except for floss use (Q7) fulfilled the criteria of *p* < 0.20 to be included into the prediction modelling.

**Table 2:** Responses to the self-reported oral health (SROH) questionnaire, and their individual, unadjusted associations with periodontitis

Self-reported oral health item	Response, n (%)	Individual unadjusted odds ratio [95% CI]			
		Total periodontitis (moderate or severe)	p-value	Severe periodontitis	*p*-value
Q1. Gum disease					
Yes^b^	54 (34.6)	3.483 [1.553–7.813]	0.002*	3.769 [1.775–8.000]	< 0.001*
No	86 (55.1)				
Don’t Know^a^	15 (9.6)				
Missing^a^	1 (0.6)				
Q2. Own teeth/gum health					
Poor^c^	38 (24.4)	6.457 [3.087–13.502]	< 0.001*	4.607 [1.974–10.751]	< 0.001*
Fair^c^	61 (39.1)				
Good	44 (28.2)				
Very good	10 (6.4)				
Excellent	2 (1.3)				
Don’t Know^a^	1 (0.6)				
Q3. Gum treatment					
Yes^b^	26 (16.7)	4.658 [1.327–16.348]	0.010*	4.558 [1.883–11.032]	< 0.001*
No	127 (81.4)				
Don’t Know^a^	3 (1.9)				
Q4. Loose teeth					
Yes^b^	38 (24.4)	12.783 [2.938–55.606]	< 0.001*	4.929 [2.267–10.714]	< 0.001*
No	118 (75.6)				
Q5. Bone loss					
Yes^b^	17 (10.9)	9.195 [1.184–71.430]	0.011*	3.590 [1.276–10.103]	0.011*
No	137 (87.8)				
Don’t Know^a^	2 (1.3)				
Q6. Tooth appearance					
Yes^b^	57 (36.5)	2.108 [1.006–4.417]	0.046*	2.483 [1.244–4.954]	0.009*
No	99 (63.5)				
Q7. Floss use					
1–7 days/wk.^b^	115 (73.8)	1.837 [0.871–3.872]	0.108*	1.406 [0.636–3.108]	0.398
Never	40 (25.6)				
Missing^a^	1 (0.6)				
Q8. Mouthwash use					
1–7 days/wk.^b^	51 (32.7)	1.658 [0.787–3.492]	0.181*	1.988 [0.987–4.004]	0.053*
Never	105 (67.3)				

^a^The response ‘Don’t know’ and missing values were excluded for further analysis, similar to Eke et al. [14]

*p*-values from chi-square tests

^b^reference category

^c^Combined reference category, according to Eke et al. [14]

*sufficing the cut-off *p*-value ≤0.20, making the predictor suitable for binary logistic regression modelling

### Salivary biomarkers

**Fig. 1:**
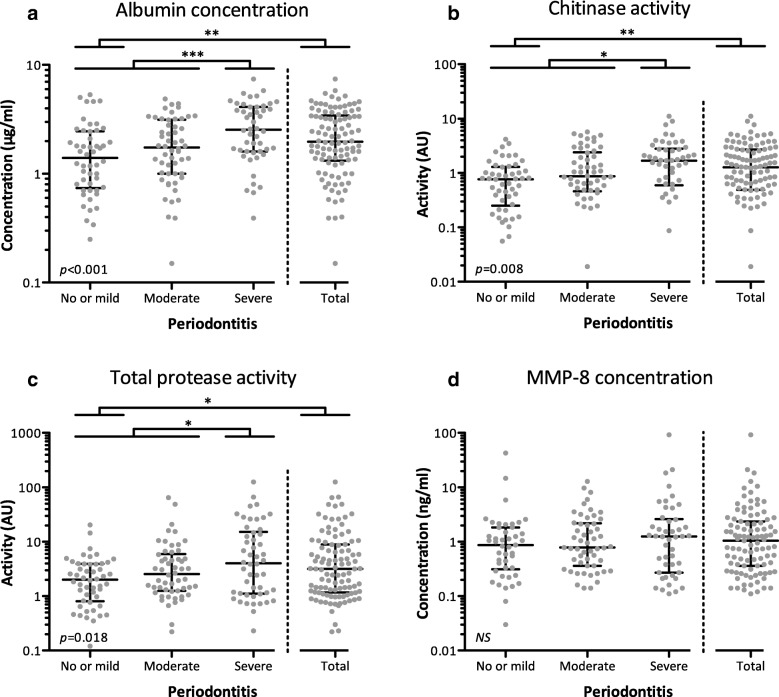
Biomarkers in oral rinse samples. (**a**) Albumin concentration (μg/ml); (**b**) Chitinase activity (AU); (**c**) Total protease activity (AU); (**d**) MMP-8 concentration (ng/ml). Concentrations and activities of the biomarkers are presented on a logarithmic scale (y-axis), separated for each periodontitis classification (x-axis). Total periodontitis combines moderate and severe cases. Each dot represents one patient, the horizontal bars in each graph display the medians and interquartile ranges (IQR). Differences across the three periodontitis classification groups (no/mild, moderate and severe periodontitis) were analyzed using Kruskal-Wallis tests, of which the *p*-value is presented in the bottom left corner of each graph. By using Mann-Whitney U tests, patients with total periodontitis were compared with patients with no/mild periodontitis; those with severe periodontitis were compared with patients without severe periodontitis. *p < 0.05, **p < 0.01, ***p < 0.001; *AU* Arbitrary Unit, *NS* Not Significant

Figure 1 shows biomarker concentrations or activities for the different groups of periodontitis severity (For exact data, see Additional file 2). Albumin concentration, chitinase activity and protease activities were significantly different across all three periodontitis groups (*p* < 0.001, *p* = 0.008 and *p* = 0.018, respectively). In univariate analyses, when comparing total periodontitis with mild/no periodontitis, albumin concentration, chitinase activity and protease activity were all significantly increased. Similar results were observed when patients with severe periodontitis were compared with dental patients without severe periodontitis. In contrast, MMP-8 concentrations did not show an association with any form of periodontitis, and did also not suffice the norm of *p* < 0.20 to be included in the binary logistic regression modelling (*p* = 0.325 for total and *p* = 0.504 for severe periodontitis).

### Prediction modeling

#### Step 1: modeling

None of the predictors showed multicollinearity, as tolerance values ranged from 0.64 to 0.87 (≥0.1) and VIF values ranged from 1.15 to 1.56 (≤5). Table 3 (total periodontitis) and Table 4 (severe periodontitis) list which predictors remained in the final prediction models after stepwise backward removal of non-significant variables. The reference category and weight (B) are also provided for each predictor.

**Table 3:** Logistic regression models for predicting total periodontitis (moderate and severe combined) and their performance

Predictor	Model 1: Questionnaire, demographic data and biomarkers			Model 2: Questionnaire and demographic data			Model 3: Questionnaire only		
	Contributing to model	Reference category	B	Contributing to model	Reference category	B	Contributing to model	Reference category	B
Predictor									
Q1. Gum disease									
Q2. Own teeth/gum health	+	Negative	1.800	+	Negative	1.692	+	Negative	1.686
Q3. Gum treatment	+	Yes	1.367	+	Yes	1.286	+	Yes	1.584
Q4. Loose teeth	+	Yes	1.774	+	Yes	1.560	+	Yes	1.969
Q5. Lost bone									
Q6. Tooth appearance									
Q7. Floss use									
Q8. Mouthwash use	+	1–7 days	1.005	+	1–7 days	1.075	+	1–7 days	0.846
Age (years)	+	> 39	2.206	+	> 39	2.209			
Sex									
Smoking									
Albumin concentration									
Chitinase activity	+	n/a	0.394						
Protease activity	+	n/a	0.094						
Model performance									
AUROCC (95% CI)	0.91 (0.86–0.96)			0.88 (0.82–0.93)			0.81 (0.74–0.88)		
Predicted probability cut-off	0.662			0.678			0.623		
Hosmer-Lemeshow	0.777			0.910			0.937		
Sensitivity (95% CI), (%)	80 (71–87)			78 (69–86)			85 (78–92)		
Specificity (95% CI), (%)	88 (76–95)			84 (71–93)			63 (49–76)		
PPV (95% CI), (%)	93 (86–97)			91 (84–95)			82 (75–89)		
NPV (95% CI), (%)	69 (59–77)			66 (57–74)			68 (55–81)		

The table lists all candidate predictors (data presented in Table 1, Table 2, and Fig. 1). The predictors marked with a + are the ones that remained in the prediction model after stepwise backward regression modeling (see Methods section of main text). The reference category represents the response which was coded 1 in the analysis. B is the regression coefficient of the predictor, indicating its weight.

*n/a* not applicable, *AUROCC* area under receiver operator characteristic curve, *PPV* positive predictive value, *NPV* negative predictive value

**Table 4:** Logistic regression models for predicting severe periodontitis and their performance

Predictor	Model 1: Questionnaire, demographic data and biomarkers			Model 2: Questionnaire and demographic data			Model 3: Questionnaire only		
	Contributing to model	Reference category	B	Contributing to model	Reference category	B	Contributing to model	Reference category	B
Predictor									
Q1. Gum disease	+	Yes	1.573						
Q2. Own teeth/gum health							+	Negative	1.152
Q3. Gum treatment	+	Yes	2.100	+	Yes	2.073	+	Yes	2.235
Q4. Loose teeth				+	Yes	1.277	+	Yes	1.306
Q5. Lost bone									
Q6. Tooth appearance				+	Yes	1.590	+	Yes	0.973
Q8. Mouthwash use	+	1–7 days	1.745	+	1–7 days	1.440	+	1–7 days	1.181
Age (years)	+	> 39	1.455	+	> 39	1.615			
Sex	+	Male	1.272	+	Male	1.091			
Smoking	+	Yes	2.007						
Albumin concentration	+	n/a	0.727						
Chitinase activity									
Protease activity									
Model performance									
AUROCC (95% CI)	0.89 (0.85–0.95)			0.82 (0.75–0.89)			0.78 (0.71–0.86)		
Predicted probability cut-off	0.250			0.214			0.273		
Hosmer-Lemeshow	0.827			0.963			0.717		
Sensitivity (95% CI), (%)	86 (71–95)			80 (66–90)			65 (52–79)		
Specificity (95% CI), (%)	78 (68–86)			70 (60–79)			81 (73–88)		
PPV (95% CI), (%)	62 (52–71)			56 (48–64)			62 (48–75)		
NPV (95% CI), (%)	93 (86–97)			88 (81–93)			83 (76–90)		

The table lists all candidate predictors (data presented in Table 1, Table 2, and Fig. 1). The predictors marked with a + are the ones that remained in the prediction model after stepwise backward regression modeling (see Methods section of main text). The reference category represents the response which was coded 1 in the analysis. B is the regression coefficient of the predictor, indicating its weight

*n/a* not applicable, *AUROCC* area under receiver operator characteristic curve, *PPV* positive predictive value, *NPV* negative predictive value

#### Step 2: discrimination

**Fig. 2:**
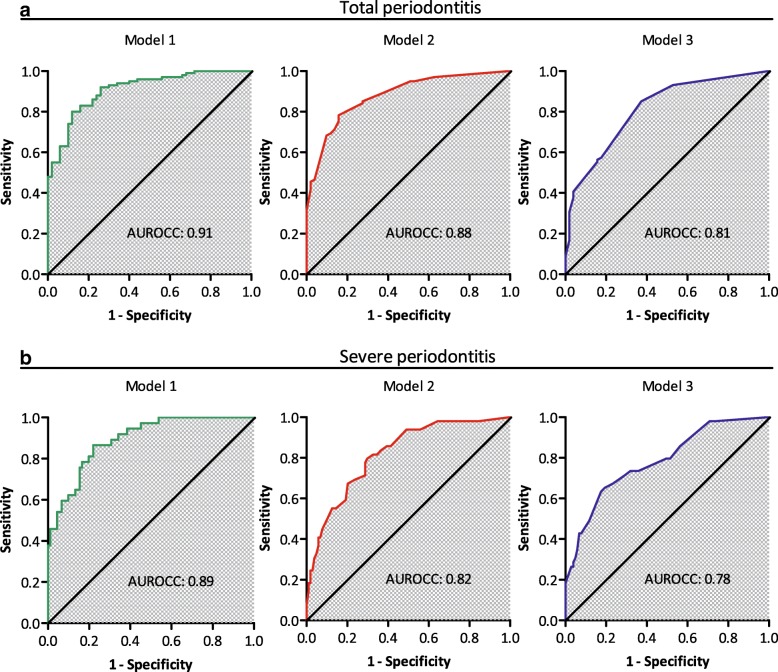
ROC curves of the prediction models. The Receiver Operating Characteristic (ROC) curves for each prediction model. The diagonal line represents the situation where the model doesn’t makes decisions better than “random” (i.e. flipping a coin), and therefore has no discriminative value. In each graph, the area under the ROC curve (AUROCC) is given, which is a measure for the discriminative performance of the model. Panel (**a**) represents the three models predicting total periodontitis (moderate and severe combined). The lower panel (**b**) shows the models predicting severe periodontitis. Model 1: self-reported oral health (SROH) questionnaire, demographics and biomarkers. Model 2: SROH questionnaire and demographics. Model 3: SROH questionnaire only

ROC curves for the prediction models are shown in Fig. 2. For total periodontitis, the AUROCC was 0.91 for model 1 (SROH, demographics and biomarkers), 0.88 for model 2 (SROH and demographics) and 0.81 for model 3 (SROH only). For severe periodontitis, this was 0.89 (model 1), 0.82 (model 2) and 0.78 (model 3). The coordinates of the ROC curves also produced the predicted probability cut-off values for each model, shown in Tables 3 and 4.

#### Step 3: calibration

Analysis of the Hosmer-Lemeshow test for the total periodontitis models revealed a goodness of fit of 0.777 for model 1, 0.910 for model 2 and 0.937 for model 3 (Table 3). For severe periodontitis, this was 0.827 for model 1, 0.963 for model 2 and 0.717 for model 3 (Table 4). The fact that none of these tests resulted in *p*-values below 0.10 indicates that each model showed an adequate goodness of fit.

#### Step 4: clinical values

Two-by-two contingency tables, with predicted disease outcome versus the actual disease state, revealed clinical values for each model. These values – sensitivity, specificity, positive predictive value (PPV) and negative predictive value (NPV) – can be found in Table 3 (total periodontitis) and Table 4 (severe periodontitis).

#### Step 5: scoring method

The following algorithms – that were used to calculate an individual sumscore for each patient – were emerged from the modelling:

Total periodontitis:

$$ Model\ 1=1.800\ast Q2+1.367\ast Q3+1.774\ast Q4+1.005\ast Q8+2.206\ast Age+0.394\ast Chitinase+0.094\ast Protease $$

$$ Model\ 2=1.692\ast Q2+1.286\ast Q3+1.560\ast Q4+1.075\ast Q8+2.209\ast Age $$

$$ Model\ 3=1.686\ast Q2+1.584\ast Q3+1.969\ast Q4+0.846\ast Q8 $$

Severe periodontitis:

$$ Model\ 1=1.573\ast Q1+2.100\ast Q3+1.745\ast Q8+1.455\ast Age+1.272\ast Sex+2.007\ast Smoking+0.727\ast Albumin $$

$$ Model\ 2=2.073\ast Q3+1.277\ast Q4+1.590\ast Q6+1.440\ast Q8+1.615\ast Age+1.091\ast Sex $$

$$ Model\ 3=1.152\ast Q2+2.235\ast Q3+1.306\ast Q4+0.973\ast Q6+1.181\ast Q8 $$

By using the predicted probability cut-off value, derived using the ROC charts (step 2), the corresponding threshold sumscore could be determined. For total periodontitis, these threshold sumscores were 4.03 for model 1, 3.25 for model 2 and 1.68 for model 3. For severe periodontitis, this was 5.94 for model 1, 3.69 for model 2 and 2.48 for model 3. Therefore, depending on the model used, any obtained sumscore exceeding these threshold values indicate that the individual screens positive for periodontitis.

The screening tool for total periodontitis, based on the algorithms of model 2, is freely accessible at www.perioscreening.com. This only requires completing the SROH questionnaire and entering demographic data.

## Discussion

With this study, we developed and assessed several prediction models, which provide primary care professionals with an easy, non-invasive clinical tool to screen for periodontitis. The models that used SROH in combination with demographics and biomarkers performed best. These models showed high discriminative value (AUROCC of 0.91 for total and 0.89 for severe periodontitis) and effectiveness (sensitivity and specificity of 80 and 88% for total periodontitis, and 87 and 78% for severe periodontitis).

To our knowledge, this is the first study to combine SROH and salivary biomarkers to predict the presence of periodontitis. Importantly, the sampling method used in this study to collect oral fluid for biomarker analysis – a ‘quick and easy’ oral rinse protocol – appeared to be effective and feasible. Moreover, the choice to use saline rather than PBS prevented a bitter taste for the patient. One could consider our findings as a proof of concept that an oral rinse with unbuffered saline solution can also detect differences in enzyme activity and protein concentrations across healthy subjects and periodontitis patients. It should also be noted that the “ionic strength” of both saline and PBS is comparable, hence no differences in preservation of proteins and enzyme activity (such as protease activity) are to be expected. This indicates that oral rinse samples with saline could be a useful and user-friendly addition to the daily practice of family physicians, diabetologists, cardiologists and other non-dental medical professionals who have a need to screen for periodontitis. Also, future studies within a primary care setting could consider using oral rinse samples, rather than complex and time-consuming whole saliva and gingival crevicular fluid sampling methods.

The finding that albumin concentration was increased in patients with total and severe periodontitis (Fig. 1, panel A) is in agreement with previous research [28, 29]. It is suggested that this increase reflects leakage of plasma proteins into the oral cavity, caused by inflammation [28]. This would explain why the mean albumin concentration was particularly high in patients with severe periodontitis, which is generally characterized by active inflammatory processes, often reflected by increased gingival bleeding [30]. Indeed, patients with severe periodontitis had a higher mean percentage of bleeding on probing compared to patients without severe periodontitis (Table 1), indicating active inflammation and thereby increased leakage of proteins such as albumin. The observed increase in chitinase activity in patients with total and severe periodontitis (Fig. 1, panel B) has also been demonstrated in previous research [21]. Interestingly, improvement of periodontal health after periodontal treatment resulted in a significant decrease of chitinase activity [31]. It is suggested that salivary chitinase acts as a defense mechanism against chitin-containing micro-organisms in the oral cavity, such as pathogenic yeasts [32]. The higher chitinase activity in periodontitis patients can be explained by a higher salivary bacterial load, compared to controls [33], as well as by the fact that chitinase activity has been found to be elevated in other chronic inflammatory diseases, such as Crohn’s disease, asthma or hepatitis [34, 35]. Chitinase activity did contribute to the prediction of total periodontitis in the present study, but not to the prediction of severe periodontitis. Protease activity was not only increased in patients with periodontitis (Fig. 1, panel C), it also remained in the prediction model for total periodontitis. A recent study showed similar results, as protease activity in patients with periodontitis was more than 3 times higher, compared to healthy controls [36]. The only biomarker in the current study not associated with periodontitis was MMP-8 (Fig. 1, panel D). This result was rather surprising, since it is generally accepted in literature that salivary MMP-8 is increased in patients with periodontitis [17, 37, 38]. We acknowledge that analyzing active forms of MMP-8 rather than protein concentration in saliva could have been an alternative choice, since several studies found that these active forms could distinguish periodontally diseased subjects [37, 39, 40]. However, the added value of using the active form of MMP-8 could be relatively small, as our findings suggest that in general, salivary biomarkers might be redundant in periodontitis screening. Although omitting the biomarkers from the models did reduce the accuracy to some extent, it was demonstrated that the SROH questionnaire alone still performed very well (total periodontitis: AUROCC = 0.81, sensitivity = 85%, specificity = 63%; severe periodontitis: AUROCC = 0.78, sensitivity = 65%, specificity = 81%). This is particularly interesting considering the minimal effort it requires to ask the eight questions. Asking Q2, Q3, Q4 and Q8 from the SROH questionnaire was sufficient to predict total periodontitis in the current setting of the ACTA dental school, and by adding Q6, severe periodontitis could also be predicted with relatively good accuracy. This implies that, even in a setting without the possibility of collecting oral rinse samples, it is possible to predict the presence of (severe) periodontitis with relatively high accuracy.

Our prediction models performed remarkably similar to those developed in the United States and France. In the study from the United States, the best performing model, consisting of five SROH questions and demographics, could also predict periodontitis with relative high accuracy (sensitivity: 85%, specificity: 58%, area under the ROC curve [AUROCC]: 0.81) [14]. In the study from France, a sensitivity of 78.9%, a specificity of 74.8% and an AUROCC of 0.821 were achieved when combining SROH and demographics [15]. This indicates that, provided that more studies are performed in various parts of the world, the SROH questionnaire could be a globally useful screening tool.

A major strength of this study was the fact patients were included consecutively, without any prior knowledge of their oral health status. Also, the periodontal examination provided us with what is considered the golden standard for periodontal disease diagnosis, a crucial aspect for internal validation of a screening tool. The original definition by the CDC-AAP was used to define periodontitis cases [20], rather than the updated version [41]. In the updated version, total periodontitis – besides moderate and severe periodontitis – also includes mild periodontitis (≥2 interproximal sites with attachment loss of ≥3 mm, and ≥ 2 interproximal sites with pocket depth of ≥4 mm [not on the same tooth] or one site with pocket depth ≥ 5 m) [41]. Even though we acknowledge that, formally, these patients are considered cases as well, it was beyond the purpose of our screening tool to distinguish individuals with such a mild form of periodontitis. Recently, a new periodontitis classification system was proposed [42]. However, this classification system was not used in the current study, as the original questionnaire – which was the literal source of our questions – was developed and validated using the CDC-AAP periodontitis-definition.

As a rule of thumb, developing a prediction model requires at least 10 events per predicting variable (EPV), although it is suggested that this might be too conservative, and 5–9 EPV can be sufficient as well [43]. For total periodontitis, four predictors from the SROH questionnaire were complemented by one demographic variable and two salivary biomarkers, resulting in seven predictors that contributed to the model. Since the smallest groups consisted of 51 patients (no/mild periodontitis), this was sufficient to fulfill the 5–9 EPV rule [43]. This was also the case when distinguishing severe periodontitis cases (*n* = 51) from the other individuals in the study population, as seven predictors remained in that model (three questions, three demographic variables and one biomarker) [43].

Similar to observations in previous research, the prevalence of (severe) periodontitis in this study was higher with increased age, and was higher in males and smokers [44]. However, the prevalence of total periodontitis (almost 70%) and severe periodontitis (almost 35%) in our population was much higher than previous studies have shown (approximately 40% for total and 8–11% for severe periodontitis [3, 4]). Literature shows that, for severe periodontitis, prevalence and incidence generally increase with age, with a peak around the age of 38 years [2]. After this peak (i.e. in individuals of approximately 40 years or older), prevalence remains fairly constant at approximately 25–30%. However, prevalence of severe periodontitis was already more than 30% in our total population, and even almost 45% in patients aged 38 or older. Therefore, the population attending ACTA’s outpatient clinic is not likely to be representative of the national and global average. This might affect the models’ performance when it is implemented in practice. External validation is therefore recommended.

The final results from this study provide screening tools for two specific disease outcomes, namely total and severe periodontitis. Before actual implementation into practice can be realized, one needs to decide for which disease outcome the tool will be used. When considering the biologic relationship between periodontitis and general health, severe periodontitis seems to be the disease outcome of most interest. For example, periodontitis is associated with the development of diabetic complications in a dose-dependent relationship: particularly severe periodontitis increases the risk for complications [9, 45]. However, when observing the performances of the models, those for total periodontitis appear to perform slightly better. Also, by screening for total periodontitis, most severe cases will still be identified. Importantly, the screening tool only gives an indication; the actual periodontal diagnosis still has to be made by a dentist. Therefore, preferring to screen for total periodontitis rather than severe periodontitis seems to be justifiable. The screening tool for total periodontitis, based on the algorithms including SROH and demographics, is freely accessible at www.perioscreening.com.

## Conclusions

In conclusion, within the limitations of this study, prediction models for periodontitis – based on self-reported oral health, demographics and/or biomarkers – proved to be feasible and accurate. The current findings corroborate and extend those from the United States and France, and provide primary care physicians and medical specialists with the required, user-friendly tool to screen for periodontitis. Implementation of such a tool in daily practice could support medical professionals in their responsibility of monitoring oral health of their patients, and urging them to visit a dentist for periodontal diagnosis and when indicated to undergo proper treatment.

## Additional files

Additional file 1: Self-reported oral health questions and Dutch translations. This additional file presents the self-reported oral health questions, their abbreviations and the Dutch translations. (DOCX 14 kb)

Additional file 2: **Table S1.** Biomarkers in oral rinse samples. This additional file present median biomarker concentrations or activities for the different groups of periodontitis severity, supplementary to Fig. 1. (DOCX 14 kb)

## Abbreviations

ACTA: Academic centrum Tandheelkunde Amsterdam (Academic Center for Dentistry Amsterdam)
ANOVA: Analysis Of Variance
AUROCC: Area under receiver operator characteristic curve
CDC-AAP: Centers for disease control and prevention-American academy of periodontology
ELISA: Enzyme-Linked Immuno Sorbent Assay
EPV: Events per predicting variable
IDF: International diabetes federation
IFMA: Immunofluorometric assay
MMP-8: matrix metalloproteinase-8
NHG: Nederlandse Huisartsen Genootschap (Dutch College of General Practitioners)
NPV: Negative predicative value
PBS(−t): phosphate buffered saline (−tween)
PPV: Positive predictive value
SROH: Self-reported oral health
VIF: Variance inflation factor
