# Supplementary material for: A rapid, non-invasive tool for periodontitis screening in a medical care setting
Source: BMC Oral Health. 2019 May 23;19:87. doi: 10.1186/s12903-019-0784-7 (PMC6533660; doi:10.1186/s12903-019-0784-7)
Supplement: Supplementary file 1 — Self-reported oral health questions and Dutch translations. This additional file presents the self-reported oral health questions, their abbreviations and the Dutch translations. (DOCX 14 kb) [file 12903_2019_784_MOESM1_ESM.docx]

| **Question number** | **Abbreviation** | **Questions and Dutch translations (italic)** |
| --- | --- | --- |
| Q1. | Gum disease | Do you think you might have gum disease?  *Denkt u dat u een tandvleesaandoening heeft, ook wel gingivitis of parodontitis genoemd?* |
| Q2. | Own teeth/gum health | Overall, how would you rate the health of your teeth and gums?  *Hoe zou u over het algemeen genomen de gezondheid van uw tanden en tandvlees beoordelen?* |
| Q3. | Gum treatment | Have you ever had treatment for gum disease such as scaling and root planing, sometimes called “deep cleaning”?  *Bent u wel eens voor een tandvleesaandoening behandeld, soms ook wel “diep schoonmaken” of “pocket behandeling” genoemd?* |
| Q4. | Loose | Have you ever had any teeth become loose on their own, without an injury?  *Heeft u wel eens last van losstaande tanden gehad, zonder dat daar een ongeluk of trauma aan vooraf ging?* |
| Q5. | Lost bone | Have you ever been told by a dental professional that you lost bone around your teeth?  *Heeft een tandheelkundige specialist u wel eens verteld dat u botverlies heeft rondom uw tanden?* |
| Q6. | Tooth appearance | During the past three months, have you noticed a tooth that doesn’t look right?  *Heeft u de afgelopen drie maanden wel eens gemerkt dat een tand er niet goed uit zag?* |
| Q7. | Floss use | Aside from brushing your teeth with a toothbrush, in the last seven days, how many times did you use dental floss or any other device to clean between your teeth?  *Hoe vaak heeft u de laatste zeven dagen, afgezien van tandenpoetsen met een normale tandenborstel, geflost of op een andere manier tussen uw tanden schoongemaakt?* |
| Q8. | Mouthwash use | Aside from brushing your teeth with a toothbrush, in the last seven days, how many times did you use mouthwash or other dental rinse product that you use to treat dental disease or dental problems?  *Hoe vaak heeft u de laatste zeven dagen, afgezien van tandenpoetsen met een normale tandenborstel, mondspoelmiddel of een ander spoelproduct gebruikt om tandheelkundige ziekte of klachten te behandelen?* |
