# Supplementary material for: A rapid, non-invasive tool for periodontitis screening in a medical care setting
Source: BMC Oral Health. 2019 May 23;19:87. doi: 10.1186/s12903-019-0784-7 (PMC6533660; doi:10.1186/s12903-019-0784-7)
Supplement: Supplementary file 2 — Table S1. Biomarkers in oral rinse samples. This additional file present median biomarker concentrations or activities for the different groups of periodontitis severity, supplementary to Fig. 1. (DOCX 14 kb) [file 12903_2019_784_MOESM2_ESM.docx]

| **Biomarker** | **No or mild**  **periodontitis**  **(n=51)** | **Moderate**  **Periodontitis**  **(n=54)** | **Severe**  **periodontitis**  **(n=51)** | **Total periodontitis**  **(moderate + severe) (n=105)** |
| --- | --- | --- | --- | --- |
| Albumin (µg/ml) | 1.50 (0.81-2.50) | 1.70 (1.00-3.15) | 2.59 (1.66-4.18)^b^*** | 2.00 (1.35-3.45)^a^** |
| Chitinase activity (AU/ml) | 0.76 (0.24-1.34) | 0.85 (0.45-2.17) | 1.54 (0.53-2.69)^b^* | 1.09 (0.48-2.55)^a^** |
| Protease activity (U/ml) | 2.30 (1.24-3.94) | 2.42 (1.24-5.31) | 2.83 (1.05-13.0)^b^* | 2.51 (1.14-7.73)^a^* |
| MMP-8 (ng/ml) | 0.87 (0.31-1.83) | 0.78 (0.36-2.17) | 1.24 (0.27-2.71) | 1.04 (0.34-2.45) |
| Data are presented as median (interquartile range).  a: significantly different from no/mild periodontitis  b: significantly different from patients without severe periodontitis  Comparisons between any two ‘diseased’ groups (severe and total periodontitis) and ‘non-diseased’ groups (non-severe and no/mild periodontitis) were performed using Mann-Whitney U tests.  *p <0.05, **p <0.01, ***p <0.001 | | | | |
